# Supplementary material for: The burden of catastrophic and impoverishing health expenditure in Armenia: An analysis of Integrated Living Conditions Surveys, 2014–2018
Source: PLOS Glob Public Health. 2022 Oct 3;2(10):e0000494. doi: 10.1371/journal.pgph.0000494 (PMC10021688; doi:10.1371/journal.pgph.0000494)
Supplement: Attachment — Submitted filename: Responses to reviewer comments_04.24.2022.docx [file pgph.0000494.s002.docx]

**Responses to Reviewer Comments (April 24, 2022)**

| **#** | **Comments** | **Response to comments** |
| --- | --- | --- |
|  | Reviewer 1 | |
| 1. | This manuscript presents information on CHE and impoverishment for Armenia. The analysis is appropriate, if only standard. I do not generally see the point of bivariate analysis, but am not generally bothered by the analysis. | Thanks for this comment. We have decided to retain the bivariate analysis to show the unadjusted association of each variable with the outcome variable for the general reader. |
| 2. | However, I find the introduction to be out-of-touch with the actual research, while the abstract and conclusion also offer discussion that seems unrelated to the analysis. Roughly speaking, I would like to see more effort go into the broad literature related to this sort of analysis. SDGs and thoughts on a program that have not really been implemented, at least by the time the data was collected, are not really appropriate | We have removed the broader SDG discussion from the abstract, introduction, and conclusion. The manuscript instead reflects on catastrophic and impoverishing health spending globally.  We have also provided an overview of Armenia’s basic benefits package and the design implications it has for out-of-pocket payments.  We also shift the rationale for this study to focus on filling the gap in the country-specific factors associated with catastrophic and impoverishing health spending in Armenia. |
| 3. | For example, in the abstract, "Preventing the burden of catastrophic ... , and other health reforms." Yet, the analysis offers no control on the relevant program. Thus, the conclusion is complete speculation, based on other literature. In that regard, the analysis simply does not tie to the "story" in the paper, and that absolutely needs to change. | This has been revised to focus on CHE and impoverishment (see authors’ response to Comment 2) whilst highlighting gaps in data that was included.  The abstract’s conclusion now reads,  ‘This detailed analysis offers an updated and nuanced insight into the trends in Armenia’s financial risk protection against catastrophic and impoverishing health expenditures and the groups this burden largely affects. The incidence of CHE and impoverishment in Armenia remains high and characterized by significant inequalities. Armenia should consider different mechanisms such as subsidizing medication and hospitalization costs for the poorest to alleviate the burden of OOP. Furthermore, even though this study did not include transport costs, it is anticipated that transport costs may further exacerbate the incidence of CHE and impoverishment in Armenia. Future analyses should include these costs.’ |
| 4. | The first paragraph in the intro is about SDGs and pooling. Yet, the analysis is not. Thus, this is not particularly relevant. The focused discussion about Armenia (second paragraph) seems a better place to start, as it is directly about the actual paper. However, in that paragraph, there is a sentence, "... - found that 16% of OOP payments exceeded 10% of annual household consumption..." It does not come through cleanly, maybe something about 16% of households? | The paragraphs have been revised to focus on financial risk protection.  The statement has been revised to reflect that 16% of Armenian households incurred CHE using the 10% of annual household consumption threshold. |
|  | **Reviewer 2** | |
| 5. | “Using logistic regression models, data from Armenia’s Integrated Living Conditions 2014 – 2018 was used to assess the incidence of catastrophic health spending and impoverishment.” – logistic regression models are assessing the risk, not the incidence. Incidence is just a descriptive statistic | Thanks for the comment.  The logistic regression models were not used to examine incidences but used to examine the determinants of CHE and Impoverishment. These were expressed in terms of Odds Ratios both unadjusted and adjusted.  The statement in the abstract has been revised. |
| 6. | The description of coverage policy and spending levels is useful for providing context, but could go more into depth. When did the BBP begin? More could be said about the depth of coverage. | Thanks for the suggestion. We have included greater detail on the history of the BBP and the depth of its coverage for context in the introduction section of the manuscript. |
| 7. | Also for how many households (ie what % of households with inpatient spending) were results imputed? | 10.8% had missing data on outpatient expenditure although they had information on number of inpatient visits over the last one year. For this group, we imputed the missing values by multiplying the median bed day costs and the average length of stay in the hospital and the no of visits in the past year, to annuitize. |
| 8. | Headcount was said to be early on calculated as the percentage of households. But then later (e.g. line 205) the authors refer to % of population or even (line 210) the number of individuals. Which is correct (ie household weights or population weights)? | Yes, headcount was calculated as the proportion of households that incurred CHE. However, we converted these into the number of individuals by multiplying the number of households incurring CHE and the average household size in 2018.  We have now provided both the number of households and the number of individuals they represent. |
| 9. | “The two thresholds were used 134 for comparison and given the fact that there is no consensus about which of the two is the better 135 threshold for assessing CHE (15).” It is not only the thresholds that make these methods different but also the denominator. See Cylus, Thomson, Evetovits (2018) “Catastrophic health spending in Europe: equity and policy implications of different calculation methods” Bulletin of the WHO for analysis that compares financial protection methods, including these two. | Thanks for the comment. The differences between these two measures has been clarified to reflect a distinction in the denominator (non-food versus total expenditure in the household), threshold (as a percentage of this denominator) and the fact that there remains a debate about which of the two is a better measure of CHE. |
| 10. | Are the quintiles used based on income, consumption or assets in the inequalities analysis? Are they adjusted for household composition? Do the results change using different definitions of household wealth (i.e. defining quintiles differently)? | We used an asset-based index to create quintiles following the Principal Component Analysis approach. We account for household composition.  We do not explore changes in the differences in the quintiles using other approaches due to lack of or inadequacy in data. For instance, there are lots of missingness on income and therefore could not use it as alternative. |
| 11. | Why do the models not also use 40% non-food as a dependent variable since it is calculated and presented in tables? | We used both measures (40% non-food and 10% total) to fit our logistic regression models in the revised draft. The 10 percent threshold models are reported in the results section due to three main reasons:   1. The 10% threshold resulted in a larger proportion of households incurring CHE compared to the 40% threshold (Table 2). Due to the larger population, much more variance is captured using this threshold compared to when the 40% threshold is used. 2. Both thresholds indicated a similar trend in the proportion of households incurring CHE hence factors were assumed to follow a similar trend. 3. Results for the 40% non-food expenditure threshold were similar to those of the 10% threshold (See Supplementary table 1) |
| 12. | I have a number of concerns about the explanatory variables used in the models, many of which are endogenous. For example, access to BBP and being socially vulnerable. In the adjusted models, having access to BBP is not associated with more or less risk of catastrophic spending. This underscores the difficulties in interpretation with so many endogenous variables in a model.  But the same is true with education and socioeconomic status. This would seem to make it impossible to interpret the odds ratios in the adjusted models (and indeed, even in the unadjusted models due to lots of confounding) | The explanatory variables used in this analysis were predominantly informed by literature (References 5, 16, and 17-24) and their availability in the dataset.  There is indeed a chance of endogeneity in the model however, we have now acknowledged this limitation and clarified that these findings should be interpreted as associations rather than causality.  Furthermore, a multi-collinearity check did not show any correlations between the variables. |
| 13. | Is hypertension a suitable proxy for health status? Are there other health variables in the dataset? Hypertension may also correlate with age, which itself correlates with more health care use. Likewise being in paid work is also predicted by health status, as is high socioeconomic status. I am just not sure what the unadjusted models are telling you which would be useful for understanding policy. | Based on the available variables in the ICLS dataset, hypertension represented the best proxy for health status. A multi-collinearity check did not show any correlations between the variables. |
| 14. | I’m also not sure about the impoverishign spending models given the very limited variability. Have the authors considered looking at spending for the “further impoverished” ie those who are below the poverty line and spending anything as well? This is becoming more common, even as part of global monitoring. Is $1.90 per person per day really appropriate for an upper middle income setting? One might argue for a higher poverty threshold (e.g. 60% median income even)? | We have included findings for those further impoverished in Table 3.  We have used two poverty lines the $1.90 international poverty line and the $5.50 recommended by the World Bank for upper-middle income countries such as Armenia. |
| 15. | Line 44 “as” should be “has” | The sentence has been removed as its content focused on the SDGs. |
| 16. | Line 65: “found that 16 percent of OOP payments exceeded 10 percent of annual household consumption in 2013” is unclear. I think you mean that 16% of people lived in households for whom OOP payments exceeded 10 percent of annual household consumption?? Likewise the following sentence that catastrophic spending incidence has increased by 3.3% per year does not specify what years it is referring to. | Line 65 changed.  The sentence has been revised to read as,  “The most recent examination of Armenia’s CHE found that 16 percent of Armenian households’ OOP payments exceeded 10 percent of annual household consumption in 2013 (3). With an average annual 3.3 percent increase in the incidence of catastrophic OOP payments between 2010-2013, Armenia’s incidence of catastrophic health payments has grown faster than any other country in the world (3).” |
| 17. | What explains variability in CHE and impov over time? | We have provided several reasons for the variability in CHE and impoverishment over time in the Discussion section (see lines 374-382).  Several reasons could be attributed to the changes in CHE and impoverishment. These include changes to the benefits package composition, such as services, tariffs, and qualifying groups, which in part explain changes in OOP and therefore the incidence of CHE and impoverishment.  Additionally, Armenia’s wealth has grown over time. |
| 18. | No descriptive statistics are reported for the sample. What % are eligible for BBP, for example? Where are medicines? Outpatient spending? Medicines are typically the driver of financial hardship. “CHE incidence dramatically 323 increased in 2017 - reflecting in part a fall in per capita public health spending between 2016 and 2017 324 - before dipping again in 2018.” Line 323… this is not part of the analysis as far as I can tell. | Table 1 was added to provide a summary of the surveyed household’s characteristics, including the number of households with at least one member who qualifies for the BBP.  Table 2 now shows OOP expenditures by type: Outpatient, Inpatient and the total expenditure which are calculated after combining spending on medicines, laboratory and radiology investigations, and other direct payments made to the cashier or medical staff during inpatient or outpatient visits, including informal payments  The dramatic increase in CHE in 2017 has been reported in Table 2, where irrespective of the consumption threshold, the incidence of CHE was higher in 2017 than in 2016 and the previous years for the 10% threshold.  In reference to the observation of the trend in per capita public health spending, we cited data from the WHO’s Global Health Expenditure and we amended the phrasing of the sentence to,  “Although there was a general decrease in CHE incidence among Armenian households between 2014 and 2018, CHE incidence dramatically increased in 2017 – **coinciding** with a fall in per capita public health spending between 2016 and 2017 - before dipping again in 2018 (35).” |
