# Supplementary material for: The burden of catastrophic and impoverishing health expenditure in Armenia: An analysis of Integrated Living Conditions Surveys, 2014–2018
Source: PLOS Glob Public Health. 2022 Oct 3;2(10):e0000494. doi: 10.1371/journal.pgph.0000494 (PMC10021688; doi:10.1371/journal.pgph.0000494)
Supplement: Attachment — Submitted filename: Response to Reviewers 08.29.2022.docx [file pgph.0000494.s003.docx]

**Responses to Reviewer Comments (August 29, 2022)**

| **#** | **Comments** | **Response to comments** |
| --- | --- | --- |
|  | Reviewer 3 | |
| 1. | (pp. 12-13, Table 2 and discussion). The authors discuss trends in OOP spending over time. However, no correction is made for inflation. Could they redo the Table and the associated discussion after appropriate changes have been made, to convert the OOP numbers to constant prices? | We have done the corrections and calculated all OOP expenditures in 2018 constant prices using consumer price index data from the world bank. |
| 2. | (pp. 17): The sub-section is titled “Determinants of CHE and impoverishing expenditure.”  In their response to reviewers the authors agreed with the reviewer and stated that they have changed their language from implying causal relationships to that of association. The heading of this section suggests that they have not made the stated correction. Please go over the manuscript carefully and make other changes as necessary. | The heading has now been changed from “Determinants of CHE and impoverishing expenditure” to “**Correlates of CHE and impoverishing health expenditure”** to indicate associations rather than causal relationships. |
| 3. | Tables 7 and 8 and associated discussion based on bivariate (unadjusted) associations.  Quite apart from the fact that the authors are overinterpreting the bivariate associations – I would urge them to take out the relevant columns in Tables 7 and 8 and the associated discussion related to “unadjusted” associations in its entirety. This material is simply not credible (notwithstanding Reviewer 1’s comments) and will mislead readers, esp. as a lot of the relationships vanish once we move to a multivariate framework. | Thank you for the comment. We however think that it may be important to retain the bivariate analysis results in this study for the following reasons:   a) Bivariate associations indicate the level of association between the independent variable and the outcome variable and often in as much as some may tend to be significantly associated, they end up not being significant factors in multivariable analysis. We think it is very important to show these changes. b) Many other studies have presented findings in this format where both bivariate analysis and multivariable analysis results are presented, below are a couple of these studies:   Consequently, we have however removed the description of bivariate analysis in the text but retained the values in the tables. |
| 4. | Not sure I understand why receipt of remittances is used as an explanatory variable. There are only two possibilities: (1) it is a proxy for economic well-being – if so, given that you are already using a wealth index, this is not necessary. There is such a thing as too many explanatory variables (2) a more serious problem is that remittances are a response to health expenditure needs. If so, this implies a serious endogeneity problem. I would recommend dropping this variable from the list of explanatory variables. | This variable has been dropped and another model fitted excluding this variable. |
| 5. | Another variable that is potentially problematic (and endogenous) is length of hospital stay – esp. if concerns about impoverishment affect whether you get discharged early from a hospital. I would recommend dropping this variable as well | We agree with you. The length of stay variable has been removed from the models and another model fitted excluding this variable. |
